# Oxidative Stress in Poultry and the Therapeutic Role of Herbal Medicine in Intestinal Health

**DOI:** 10.3390/antiox13111375

**Published:** 2024-11-10

**Authors:** Yuan Li, Kai Wang, Chunmei Li

**Affiliations:** College of Animal Science and Technology, Nanjing Agricultural University, Nanjing 210095, China; 2021105050@stu.njau.edu.cn (Y.L.); wk@stu.njau.edu.cn (K.W.)

**Keywords:** poultry, intestine, oxidative stress, herbal medicine

## Abstract

The intensive broiler farming model has accelerated the development of the poultry farming industry. However, it has also inevitably brought about many stressors that lead to oxidative stress in the organism. The intestine is the leading site of nutrient digestion, absorption, and metabolism, as well as a secretory and immune organ. Oxidative stress in animal production can harm the intestine, potentially leading to significant losses for the farming industry. Under conditions of oxidative stress, many free radicals are produced in the animal’s body, attacking the intestinal mucosal tissues and destroying the barrier integrity of the intestinal tract, leading to disease. Recently, herbs have been shown to have a favorable safety profile and promising application in improving intestinal oxidative stress in poultry. Therefore, future in-depth studies on the specific mechanisms of herbs and their extracts for treating intestinal oxidative stress can provide a theoretical basis for the clinical application of herbs and new therapeutic options for intestinal oxidative stress injury during poultry farming. This review focuses on the causes and hazards of oxidative stress in the intestinal tract of poultry, and on herbs and their extracts with therapeutic potential, to provide a reference for developing and applying new antioxidants.

## 1. Introduction

As a globally significant source of meat and eggs, the efficiency of poultry production and the quality of its products are paramount. The livestock industry must provide healthy, eco-friendly, and sustainable animal food. With the prevalence of high-density, intensive feeding patterns, poultry are inevitably exposed to stressors from various environmental factors, like temperature, ventilation, and light, as well as nutritional factors, such as oxidized fats, mycotoxins, and heavy metals [1]. These stressors can lead to detrimental alterations at the molecular, cellular, and physiological levels, thereby diminishing the production performance of poultry. Under normal circumstances, poultry have a well-established antioxidant system, including enzymatic and non-enzymatic antioxidants. These work together to eliminate free radicals and maintain the body’s redox balance. During stress, the redox balance in animals is disrupted, which induces oxidative stress (OS) [2]. OS refers to an imbalance between oxidants and antioxidants in the body, where the production of reactive oxygen species (ROS) and other free radicals exceeds the defense capabilities of the organism. These radicals are produced as by-products during normal cell metabolic processes, such as mitochondrial respiration and immune responses [3,4].

The intestine is a highly complex and dynamically changing organ that is both the primary site of nutrient digestion and absorption and a natural barrier that protects the balance of the body’s internal environment. The location of the intestinal epithelium between the organism and the luminal environment, the rapid renewal rate of the intestinal mucosa, and its convective oxygen exchange mechanism determine that it is more susceptible to oxidative damage under stress [5,6]. Once an excess of oxidants is produced in the intestine of poultry, the body cannot rely on its antioxidant system to clear it, leading to the development of OS. Intestinal OS has been observed in various poultry diseases, including necrotizing enteritis, poultry coccidiosis, and Newcastle disease [7,8,9]. When oxidative stress occurs in the intestines of poultry, it triggers an inflammatory response that disrupts both the intestinal barrier and the microecological balance [10,11]. The direct consequence of disrupted intestinal mucosal function is a decrease in the efficacy of digestive and absorptive processes, adversely impacting the overall poultry performance.

Herbal medicines are rich in active ingredients such as polysaccharides, saponins, polyphenols, and flavonoids, which have essential roles in anti-cancer, anti-virus, anti-inflammation, and anti-oxidation activities. Recent studies have shown natural herbs’ preventive and therapeutic effects on diseases caused by OS, such as *Viola yedoensis Makino*, *Rosmarinus officinalis*, and *Artemisia ordosica* [12,13,14]. The phytochemical compounds typically reduce reactive oxygen species (ROS) levels in poultry by modulating signaling pathways such as Kelch-like ECH-associated protein 1/nuclear factor erythroid 2-related factor 2(Keap1/Nrf2), nuclear factor kappa-B (NF-κB), and phosphatidylinositol-3-kinase/protein kinase B (PI3K/Akt) [15]. A recent study utilizing a novel 3D chicken enteroid model reported that phytopharmaceuticals can enhance their anti-inflammatory and wellness properties by improving paracellular permeability, inhibiting the expression of pro-inflammatory cytokines, and reducing intracellular oxidative stress [16]. Righi et al. (2021) evaluated the efficacy of plant-based feed additives as natural antioxidants in poultry feed [17]. This demonstrates that including plant-based substances in poultry diets is a viable strategy to combat OS encountered in poultry production.

This paper provides an overview of the effects of OS on intestinal health in poultry and factors predisposing poultry to the development of OS in the intestine. It explores the therapeutic potential of herbs in alleviating OS in poultry intestines to inform the development and application of natural anti-OS active ingredients in developing and applying novel antioxidants for livestock and poultry.

## 2. Oxidative Stress and Antioxidant Defense System of Poultry

The term OS first appeared in a book entitled “oxidative stress” and is defined as “a disturbance in the prooxidant-antioxidant balance in favor of the former” [18]. The process of OS generates a plethora of ROS as oxidation intermediates, which are highly reactive molecules, including superoxide anion (O_2_^−^), hydrogen peroxide (H_2_O_2_), hydroxyl radicals (·OH) and single oxygen (^1^O_2_) [19]. These highly reactive molecules can directly target biological macromolecules like lipids, proteins, and DNA, impairing cellular structure and function. Oxidative damage in poultry can negatively affect intestinal epithelial cells and physiological functions. It can also lead to a decrease in the intestinal immune response, an increase in intestinal inflammation, as well as the destruction of intestinal barriers and the intestinal micro-ecological micro-balance, which in turn affects the nutrient absorption and utilisation efficiency of the intestinal tract and thus leads to a decrease in production performance [20,21].

Surai et al. (2019) proposed the concept of a poultry antioxidant defense network that includes three levels [20]. The first layer is constructed from three major antioxidant enzymes: superoxide dismutase (SOD), glutathione peroxidase (GPx) and catalase (CAT). SOD in poultry is tissue-specific and is influenced by various stress-related factors, such as genetics, nutrition, and toxins. The additional synthesis of SOD under stress conditions is an adaptive mechanism that reduces ROS formation and prevents OS [22]. Determination of the expression sites of antioxidant enzymes in the poultry intestine revealed that the proteins SOD1 and glutathione peroxidase (GSH-Px7) were mainly present around the basolateral and apical membranes of mature epithelial cells of the intestinal tract [23], suggesting the vital role of these antioxidant enzymes in responding to the destruction of the intestinal barrier by free radicals. The second layer is some non-enzymatic antioxidants. These antioxidants protect cells from OS damage by directly neutralizing free radicals or through other mechanisms [24]. The third level of antioxidant defense involves the repair (methionine sulfoxide reductase, heat shock proteins, DNA repair enzymes) or scavenging (phospholipases, proteasomes) of damaged molecules.

## 3. The Pivotal Mechanism of Oxidative Stress

OS involves multiple essential signaling pathways that play crucial roles in the cellular response, repair, and adaptation to oxidative damage. The Keap1/Nrf2 pathway, as one of the most effective defense systems against OS, regulates the expression of a range of protective genes, including antioxidant enzymes, detoxification enzymes, and cytoprotective proteins, which help the cell respond to OS and other harmful stimuli [25]. The NF-κB signaling pathway is extremely sensitive to redox reactions, and it has been reported that the level of ROS in the microenvironment can directly affect NF-κB activity [26]. Hydrogen peroxide is a primary source of reactive oxygen species, and it has been found that low concentrations of hydrogen peroxide specifically activate the NF-κB pathway [27]. The promoters of antioxidant enzymes such as SOD1 [28], SOD2 [29], glutathione S-transferase Pi (GST-Pi) [30], glutathione peroxidase (GPX) [31], and dimethylarginine dimethylaminohydrolase 1(DDH1) [32] contain NF-κB binding sites, and activated NF-κB can enter the nucleus to increase the transcript levels of these antioxidant enzymes. The PI3K/Akt pathway plays an essential regulatory role in the development of OS. The PI3K/Akt signaling pathway and OS can be mutually regulated by apoptosis, autophagy, senescence, and ER stress. They promote or inhibit each other [33]. Moreover, activated-state AKt can enhance adaptation to OS by inducing activation of Nrf2-related antioxidant signaling. The Wnt/β-catenin signaling pathway involves several biological processes, including embryonic development, cell proliferation, differentiation, and cancer [34]. It has been discovered that activation of this pathway can alleviate OS, which suggests a potential therapeutic approach for oxidative stress-related diseases [35].

## 4. The Influence of Oxidative Stress on the Intestinal Barrier Function of Poultry

**Figure 1:**
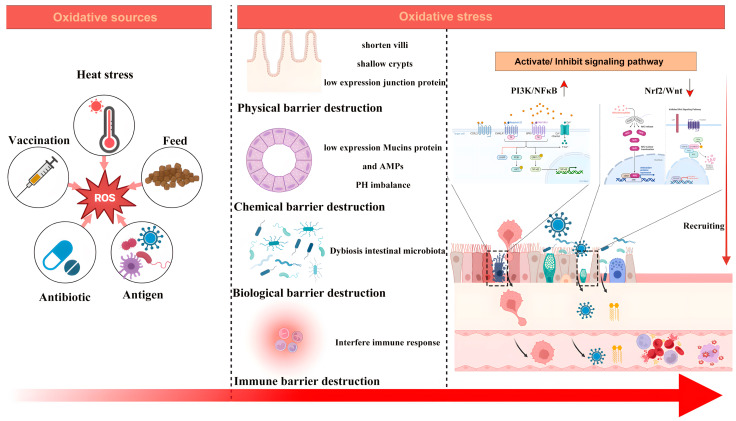
The mechanism of damage to the intestine.

As one of the most metabolically active organs in the body, the intestinal tract produces many metabolites and free radicals from its cellular metabolic processes, triggering OS. The intestinal mucosa has a high concentration of non-protein sulfhydryl groups, which are denatured and inactivated by the action of oxygen radicals on the sulfhydryl groups and thus suffer damage. Additionally, the intestine is an organ in contact with the external environment and is exposed to oxygen in the air. Oxygen can react with intracellular substances under certain conditions to form free radicals and accelerate oxidative reactions. Furthermore, dietary intake of high-fat foods and sugars and a lack of adequate antioxidants (vitamin C, vitamin E, polyphenolic compounds) may increase the intestine’s OS risk. The key factors that ensure proper intestine functioning include the morphological structure, microbiota, and immunocompetence. Figure 1 depicts the mechanism of damage to the intestine when oxidative stress occurs.

The lining of the intestine consists of a single layer of epithelial cells that are tightly packed to form a continuous barrier that covers the inner surface of the intestine. Three adhesion complexes, desmosomes, adherens junctions, and tight junctions, form a network of proteins connecting epithelial cells, preventing harmful substances and pathogens from penetrating the cellular space [36]. OS is highly susceptible to damage to the structure of the digestive tract mucosa. In vitro experimental studies showed that OS leads to tyrosine phosphorylation and induces occluding, zonula occluden-1 (ZO-1) and E-cadherin-β-catenin complexes to detach from the cytoskeleton [37]. This means that the oxides produced during OS may also disrupt the tight junctions of the gut. Excess H_2_O_2_ in the gut disrupts the cellular oxidative–antioxidant system, and the resulting ROS production alters intestinal permeability and reduces intestinal barrier function [38]. The hypoxic environment at high altitudes reduces perfusion and blood oxygen levels in animal visceral organs, triggers OS, and affects the expression of intestinal tight-junction-related proteins, thereby damaging the intestinal mucosa and leading to intestinal barrier dysfunction [39]. OS in the body due to heat stress is closely related to irreversible damage to the mitochondria. Tabler et al. (2020) showed that heat stress significantly downregulated the expression levels of the occludin, claudin-1, and ZO-1 proteins in broiler ileal tissues, ultimately increasing the intestinal permeability, with the disruption of tight junction proteins [40]. Thus, OS impairs intestinal mechanical barrier function primarily through the regulation of tight-junction-associated proteins. In contrast, pathogens as well as bacterial toxins selectively penetrate the intestinal mucosal barrier to cause damage to the organism. Therefore, the maintenance of the molecular expression of the intestinal barrier is critical for the improvement of the intestinal tract during OS.

Mucin is a macroglyco protein that coats the surface of intestinal epithelial cells, preventing the invasion of exogenous pathogens, transporting nutrients, and maintaining microbial homeostasis. The mucins *MUC2*, *MUC5AC*, and *MUC5B* in chicken intestinal tissues belong to the secreted mucins, which can be used as marker genes to determine the integrity of the intestinal mucus barrier [41]. As an environmental stress, heat stress has been shown to lead to increased OS and an imbalance in the antioxidant status of the organism. In vitro studies demonstrated that high-temperature treatment caused damage to intestinal epithelial cells by reducing the expression of tight junction proteins such as *ZO-1*, *Claudin-3*, and *Occludin*. Additionally, the transcript levels of the mucin genes MUC-2 and MUC-5AC were elevated [42]. Under heat stress, broilers exhibited increased levels of acidic mucins in both the duodenum and the ileum and mixed mucins in the ileum [43].

The number and distribution of the resident flora in the intestinal tract are relatively constant, forming an interdependent and interactive micro-ecosystem, which constitutes the biological barrier of the intestinal tract and prevents the invasion of pathogenic bacteria. The large amount of toxic ROS metabolites produced by intestinal cells can disrupt the microecological balance of the intestine by activating NF-κB, producing various cytokines and triggering an inflammatory response [44]. ROS can dysregulate pro-inflammatory pathways that regulate immune cells, leading to an imbalance in the gut microbiota [45]. The major bacterial phyla in the intestinal tract of poultry include Proteobacteria, Bacteroidetes, Firmicutes, and Actinobacteria [46]. The gut microbial diversity is a key indicator of health, and reducing this diversity can increase the risk of gut dysbiosis and related complications. Decreased gut microbial diversity promotes pathogen colonization, increasing the host’s susceptibility to disease. During the disease period in poultry, changes in microbiota abundance were primarily characterized by an increase in *Proteobacteria* and *Bacteroidetes*. Additionally, the genus *Lactobacillus*, part of the phylum *Firmicutes*, is recognized as a beneficial group for health. Former studies reported that the abundance of *Lactobacilli* and *Bifidobacteria* was found to be reduced, while the levels of *Escherichia coli*, *coliforms*, and *Clostridia* increased in the jejunal contents of heat-stressed broilers [47]. In brief, OS disrupts the structure and homeostasis of gut microbes, which may further exacerbate oxidative stress in the gut by activating inflammatory responses and producing harmful substances through the gut microbiota.

Low concentrations of free radicals are required for T-cell activation under non-stress conditions. Nevertheless, in a state of OS, free radical action produces large amounts of oxidized products in the body. These oxidized products lead to an over-activation of the immune system, which in turn causes damage to the body and manifests immunosuppression. The inflammatory response has been recognized as a mechanism of innate immunity, representing a complex biological reaction of the gut and other tissues to harmful stimuli. Macrophages and neutrophils that infiltrate the intestinal tract generate large amounts of ROS using oxidative enzymes such as nicotinamide adenine dinucleotide phosphate and other enzymes in bactericidal and pathogen elimination. These ROS help to clear the infection, but they also disrupt the integrity and tightness of the intestinal epithelium, releasing inflammatory mediators and increasing mucosal damage. Ischemic perfusion occurs when the intestine is in a state of OS, disrupting normal mitochondrial respiration and leading to an increase in the rate of glycolysis. At the same time, many toxic reactive oxygen metabolites are produced in the intestinal cells under hypoxic conditions, leading to many aggregates of inflammatory leukocytes in the ischemic region [48]. Similarly, excessive ambient temperatures impact the immune status of poultry by inducing oxidative stress and systemic inflammation [49].

## 5. Factors Inducing the Development of Intestinal Oxidative Stress in Poultry

### 5.1. Environmental Factors

High temperature is one of the most challenging environmental stresses closely related to poultry production. Broilers are thermostable animals with feathered bodies, underdeveloped sweat glands, and high metabolic heat production, making it difficult for them to dissipate heat. The appropriate ambient temperature for poultry is 16–26 °C [50], with a thermo-neutral zone that maintains the average body temperature. Poultry in this temperature range maintain good health and lower energy expenditure. However, once the temperature exceeds 26 °C, poultry adjust their physiological activities and behaviors to maintain an average body temperature to ensure their normal development. When broilers are exposed to high temperatures for long periods, heat stress is easily induced [51], leading to various problems, such as reduced feed intake, decreased performance, immunosuppression, and increased mortality, seriously affecting the efficiency of poultry farming. Early studies have found that poultry carry away excess body heat by increasing their respiration rate [52]. For every one °C increase in body temperature in poultry, the respiratory rate increases by 0.56 breaths/min. Under prolonged heat stress conditions, the continuously elevated respiratory rate reduces carbon dioxide levels. It increases the blood pH, resulting in respiratory alkalosis, which can be life-threatening for poultry [53]. As a critical mucosal barrier and nutrient digestion and absorption organ of the organism, the intestinal tract is susceptible to heat stress [54]. Broilers under conditions of persistent heat stress have reduced villus height, crypt depth, and mucosal area, which affects nutrient absorption. Studies have shown that heat stress disrupts intestinal barrier function in poultry by reducing the expression of tight junction proteins [55,56]. In addition, it also causes damage to the intestinal epithelial cells, and apoptosis increases intestinal permeability. Carbohydrates are important energy substances in poultry, involved in body metabolism and energy production, and heat stress can lead to metabolic disorders of nutrients in poultry. High temperatures activate the hypothalamic–pituitary–adrenal axis in poultry, leading to elevated plasma corticosterone levels, reducing plasma triiodothyronine concentrations and decreasing glucose utilization [57]. This endocrine dysfunction also affects the intestinal immune barrier, where pathogenic bacteria and metabolites in the intestinal lumen pass through the intestinal mucosa, inducing inflammatory infiltration and affecting nutrients’ absorption, leading to weight loss [58]. The above studies have shown that heat stress is one of the critical environmental factors inducing OS, that heat stress is closely linked to OS, and that OS is often accompanied by heat stress.

### 5.2. Feed Factors

Fats and oils in poultry feeds are high-energy nutrients that can effectively increase the energy density of feeds to meet the needs of poultry growth, production, and life-sustaining activities [59]. Not only that, the right amount of fats and oils can improve the digestibility of the feed, help poultry absorb other nutrients better, especially fat-soluble vitamins (vitamins A, D, E, and K), increase the palatability of the feed, promote the feed intake of poultry, and improve the feed utilization. Certain fats and oils (e.g., fish oils and vegetable oils) are rich in polyunsaturated fatty acids (PUFA: omega-3 and omega-6), which have a positive effect on the immune system of poultry and help to improve their resistance to pathogens [60]. However, the fats contained in the feed, especially PUFA, contain conjugated double bonds that are extremely unstable and are easily attacked by oxidizing agents and thus lose their hydrogen atoms, whereby free radicals centered on carbon atoms are formed, generating a series of lipid oxides, which increase the OS in the body [61]. Once the PUFA in feed are not adequately protected during preparation and storage, the formation of peroxides and other oxidation products can negatively affect the structure and function of the fatty acids. It may also produce toxic aldehydes, carbonyl compounds, and other volatiles, leading to decreased acceptance of the feed and affecting animal health [62]. OS in yellow-feathered broilers due to the consumption of oxidized soybean oil reduces growth performance [63]. However, Anjum et al. (2002) contended that a low concentration of oxidized oil in the diet did not impact the weight gain or feed conversion ratio in chicks [64]. The studies mentioned above have shown that the consumption of oxidized nutrient fats and oils in feeds by poultry can cause elevated levels of ROS and oxidation products in the body, leading to the development of oxidative stress, which in turn affects intestinal health and production performance.

Feed toxins are harmful substances present in animal feed that are usually produced by microorganisms (fungi and bacteria) or formed due to contamination of feed ingredients. The primary sources of feed toxins include mycotoxins, microbial contamination, and chemical contaminants [65]. Among them, fungal toxins are produced by molds, commonly those such as aflatoxin, vomitoxin, and ochratoxin [66]. Mycotoxins in poultry feed are a significant concern for animal and human safety [67]. Bacterial contamination by *Salmonella*, *Campylobacter*, *Clostridium perfringen*s, and *Escherichia coli* is a primary focus of poultry feed safety research [68,69,70,71]. Chemical contaminants contain heavy metals, pesticide residues, nitrites, and other chemicals. Mycotoxins in feed can lead to increased production of free radicals in the body by promoting inflammatory responses and cellular stress. However, they also inhibit the production and proper function of antioxidants, thereby amplifying the effects of OS [72]. Table 1 summarizes the hazards of feed toxins for poultry. It has been discovered that even very low amounts of feed toxins can trigger hazards in the intestines of poultry.

### 5.3. Microbiological Factors

Millions of microorganisms are present in the poultry gut, consisting of various bacteria, fungi, protozoa, and viruses. These microbiotas colonize various parts of the gut and contribute to digestion and absorption, immune system regulation, and resistance to pathogenic bacteria in poultry [84]. An imbalance in the gut microbiota may lead to increased amounts of microbial metabolites such as ROS, H_2_S, lipopolysaccharide (LPS), and IL, which mediate OS and inflammation [85]. Heat stress in broiler chickens is accompanied by oxidative damage in the intestine and an increased prevalence of *Escherichia coli* [86]. LPS derived from Gram-negative bacteria increases intestinal tight junction permeability, both in vitro and in vivo, through a mechanism involving TLR-4-dependent upregulation of CD14 membrane expression [87]. The intestinal barrier disruption leads to increased translocation of endotoxins in the poultry intestine, resulting in systemic inflammation [88]. Similarly, mycotoxin contamination in feed led to alterations in the cecal microbiota and increased endotoxin translocation in broiler chickens [89,90].

## 6. Plant Active Ingredients with Therapeutic Properties for the Treatment of Oxidative Stress in Poultry Intestine

A growing number of in vitro and in vivo experimental studies have confirmed that herbs and their active ingredients can be used as natural nutritional supplements to ameliorate the damage caused by OS. Various natural plant extracts and active ingredients with antioxidant and anti-inflammatory effects exist, including polysaccharides, polyphenols, peptides, organic acids, functional amino acids, and saponins. In vitro cellular models are valuable for investigating antioxidants’ protective and toxic effects. The results from these in vitro experiments often provide more intuitive insights compared to in vivo studies [91]. Table 2 summarizes the plant extracts validated by in vitro experiments as having therapeutic potential for intestinal OS damage. The preliminary assessment of the extract’s safety and efficacy can provide a foundation for future animal studies. The next section describes in detail the in vivo application of various active ingredients to anti-intestinal OS tests in animals.

### 6.1. Polysaccharides

Polysaccharides are complex carbohydrates consisting of multiple monosaccharide molecules linked by glycosidic bonds. They are widely found in plants. The main characteristics of polysaccharides include a high molecular weight and complex structure, which give them many biological functions and applications. As an organic macromolecule, the polysaccharide has antioxidant, anti-inflammatory, anti-aging, and anti-tumor effects [117]. The mechanisms by which polysaccharides exert antioxidant activity in vivo and in vitro are different. The structure of polysaccharides with antioxidant effects in vitro generally contains sulfate groups, aldehydes, hydroxyls, sulfocarbonyls, and carboxyls. These reactive groups can directly react with metal ions and free radicals, achieving scavenging effects. The antioxidant effects of polysaccharides in vitro primarily depend on their glycan ring structure, solubility, molecular weight, charge characteristics, protein molecular weight, and covalently attached phenolic compounds [118]. In vivo, polysaccharides enter the body and must be taken up by the cells, which undergo targeted transport across the cellular barrier to reach a specific location to function. After entering the body, polysaccharides undergo a series of metabolic transformations to become degraded polysaccharides in the cell and then activate the expression of downstream antioxidant enzyme genes through the regulation of signaling pathways, such as Keap1/Nrf2/ARE, which indirectly act on the role of endogenous enzymes, such as SOD, CAT, and GSH-Px [119]. Guo et al. (2024) found that *Artemisia annua* L. *polysaccharide* polysaccharides were able to maintain intestinal permeability and mucosal morphology, immune function, and antioxidant capacity by downregulating the level of *Keap1* mRNA, an intestinal Nrf2 signaling pathway, as well as upregulating antioxidant enzyme activity in broilers, thereby improving the decline in the growth performance of broilers attacked by *Escherichia coli* [120]. Luo et al. (2021) reported that *Astragalus membranaceus* polysaccharide can increase the level of sIgA in the jejunum of goslings, increase GSH-Px, and SOD activity, reduce the MDA level, reduce the expression of pro-inflammatory cytokines, and reduce the morphological damage to the jejunum, which in turn protects and alleviates the inflammatory damage to the small intestine of the goslings after plague infection [121]. Likewise, the addition of 150 mg/kg *Astragalus membranaceus* polysaccharide to the diet improved the growth performance, antioxidant function, and meat quality in broilers, and serum metabolomics analysis showed that the differential metabolites between the two groups were enriched in the glutathione metabolic pathway [122].

### 6.2. Saponins

Saponins are a class of natural compounds widely found in plants that are glycoside derivatives of steroids or triterpenoids. The molecular structure of a saponin usually consists of one or more sugar molecules and a steroidal or triterpenoidal structural unit [123]. Steroidal saponins are primarily found in Liliaceae and Dioscoreaceae, and triterpenoid saponins are mostly found in Pentacarpaceae and Umbelliferae. Much research has been conducted on the antioxidant function of saponins. Saponins are known for their antioxidant, antibacterial, and anticoccidial properties. Saponins with high antioxidant activity have been found in *Astragalus membranaceus*, *Glycyrrhiza glabra*, *Panax ginseng*, and *Urtica dioica* L. OS is commonly observed in cecal coccidiosis, and saponins may contribute to its defense by reducing ROS, thereby alleviating the lesions associated with oxidative stress [124]. Plants containing high levels of saponins can also enhance the immune system by influencing the maturation of immune organs and increasing the antibody levels in the body, resulting in a better defense against appendicitis. Ginsenosides, derived from ginseng extracts, are naturally occurring triterpenoid saponins that regulate antioxidant enzymes and help protect the intestinal barrier [125,126]. Research on the effects of ginseng extract in Caco-2 cells and broiler jejunal samples revealed that ginseng treatment reduced the expression of *HSPA1A* and the heat shock protein genes *hsp-1* and *hsp-16.2*. In contrast, the expression of the tight junction protein receptor genes *CLDN3*, *OCLN*, and *CLDN1* was upregulated under heat stress conditions [127]. In addition, Shu et al. (2021) found that dietary supplementation with *Bupleurum falcatum* L. saikosaponins helped to alleviate the reduced growth performance and delayed intestinal development, as well as the induced biological damage, including OS, impaired intestinal mucosal barrier, immune dysfunction, and apoptosis, in broiler chickens caused by NH3 exposure [128]. Similarly, the addition of *Yucca schidigera* extract containing high levels of saponins to the diet could enhance the antioxidant capacity of broilers during the fattening period by increasing the gene expression of intestinal SOD, CAT, and GPx [129].

### 6.3. Polyphenols

Polyphenols are natural secondary metabolites specific to plants, found mainly in roots, stems, leaves, and fruits, which have powerful antioxidant properties that help to neutralise free radicals, thereby reducing cell damage. Flavonoids, phenolic acids, lignans, and stilbenes are all part of the polyphenol family [15]. Polyphenols can chelate with transition metal ions, particularly Fe^2+^ and Cu^2+^, to reduce HO^•^ formation and protect the body from damage due to oxidative stress [130]. Moreover, phenoxy radicals are stable because of the benzene ring’s alkyl structure, which enhances plant polyphenols’ antioxidant capacity. Polyphenols such as catechin, lycopene, curcumin and resveratrol have been shown to enhance their antioxidant capacity by modulating the Nrf2/ARE signaling pathway and its downstream antioxidant proteins [131]. Adding *Hibiscus sabdariffa* L. extract to diets improves poultry’s metabolic function, intestinal morphology, and antioxidant capacity [132]. Carnosic acid is a phenolic diterpene found in abundance in the leaves of plants in the family Labiatae, with exceptionally high levels in dried rosemary leaves. It was found to alleviate indomethacin-induced gastric ulcer injury by decreasing the MDA and TOS levels, elevating the CAT and GPx activities, and increasing the Nrf2/HO-1 expression to reduce the OS and inflammatory responses [133]. Magnolol has been proved to linearly reduce the FCR of broilers in the first 2 weeks, increase the antioxidant capacity, and improve the intestinal tissue morphology and mucosal barrier function [134].

Flavonoids belong to an important subclass of polyphenols with a wide range of biological activities. Their structures generally contain the benzo-gamma-pyrone skeleton, which can be synthesized by various pathways, such as the shikimate, flavonoid, and phenylpropanoid pathways. According to their main structural components, their types are classified as (i) flavonoids, (ii) flavanones, (iii) flavonols, (iv) isoflavones, (v) flavanols and (vi) anthocyanins [135]. With the deepening of research, the anti-OS activity of flavonoids has received increasing attention, and their application in the anti-OS of livestock and poultry is gradually being thoroughly explored and promoted. The antioxidant mechanism of flavonoids is manifested in the following aspects. (i) Flavonoids terminate the chain reaction of free radicals by reacting with multiple hydroxyl groups in their molecules to generate stable semiquinone-type substances to scavenge ROS directly. (ii) Xanthine oxidase catalyzes the oxidation of xanthine and hypoxanthine to produce peroxide radicals. The double bond between C2 and C3 and the hydroxyl groups at the C5 and C7 positions of flavonoids have been found to inhibit xanthine oxidase activity directly. (iii) Flavonoids can exert antioxidant effects by activating the body’s inherent antioxidant system. (iv) Complexation of metal ion mechanisms [136]. One of the essential sources of free radicals is the transition metal ions in the organism, and most of these transition metal ions contain unpaired electrons, which makes it easier to catalyze the formation of free radicals. Metal ions chelate with coordination groups such as the hydroxyl and carbonyl groups in the structure of flavonoids, thus blocking the generation of free radicals in the Fenton system. Curcumin has been shown to alleviate heat stress in quail by inhibiting oxidative stress and modulating the Nrf2/HO-1 pathway [137]. Including optimized quercetin in the diet alleviates the burden on the liver and mitigates the negative effects of oxidized lipid by-products associated with a linseed oil diet [138]. *Epimedium*, a class of flavonoid-rich herbs, improves the intestinal permeability, tight junction protein function, and antioxidant capacity in broilers by altering the composition of the core intestinal flora [139]. Bamboo flavonoids, an active ingredient in bamboo leaves, have also been shown to increase the antioxidant capacity of the jejunal mucosa of broilers and improve the intestinal morphology and microbiota structure to promote the growth of broilers [140]. When applied to yellow-feathered broilers, kudzu-leaf flavonoids demonstrated the same efficacy [141].

### 6.4. Organic Acids

Organic acids are divided into natural organic acids and synthetic organic acids. Natural organic acids are biologically active organic acids extracted and separated from plants or other agricultural by-products in nature. Organic acids are a class of organic chemical substances containing carboxyl groups, which are widely found in various plant herbal medicines, such as honeysuckle, dogwood, and Kunming begonias. Many organic acids have apparent pharmacological effects, such as citric acid, malic acid, oxalic acid, and tartaric acid. Adding natural organic acids to feed can effectively improve the gastrointestinal environment of animals, making them green and residue-free [142]. Based on the previous evidence and the unique physicochemical properties of organic acids, they can be used as natural nutritional supplements to protect the intestinal tract of poultry and mitigate the impairment caused by oxidative stress. The findings of Ao et al. (2009) demonstrated that adding citric acid to poultry diets positively influenced the performance by lowering the intestinal contents’ pH, which reduced the bacteria’s tolerance to pH fluctuations [143]. Organic acids improve protein and energy digestibility by reducing the microbial competition with the host for nutrients and endogenous nitrogen loss and by reducing the production of ammonia and other growth-inhibiting microbial metabolites [144]. The research results reported by Pirgozliev et al.(2008) validated that adding fumaric and sorbic acid to broiler diets reduced the endogenous losses of *Lactobacilli* and *coliforms* in the gut [145].

### 6.5. Alkaloids

Alkaloids are nitrogen-containing essential organic compounds found in nature, characterized by their complex cyclic structures, with nitrogen atoms often integrated within the rings [146]. Exhibiting notable biological activities, bioalkaloids are considered significant active components in herbal medicines. At present, the antioxidant effect of alkaloids has been widely studied. Berberine is an isoquinoline alkaloid currently attracting significant attention due to its promising biological, therapeutic, and pharmacological activities. It can improve the immunity of broilers under a high stocking density, reduce oxidative stress, and promote the colonization of healthy microbiota in the intestinal tract, thereby improving the growth performance of broilers [147]. Recently, a study found that isoquinoline alkaloids from *Macleaya cordata* can enhance the anti-inflammatory and antioxidant capacity of broilers by regulating the TLR4/MyD88/NF-κB and Nrf2 signaling pathways, thereby reducing LPS-induced intestinal epithelial injury and intestinal dysbiosis [148]. Paraskeuas et al. (2024) confirmed that isoquinoline alkaloid blends can significantly upregulate the expression of antioxidant response-related genes in various parts of the intestine and enhance the duodenal intestinal barrier to ensure the health of broiler chickens [149]. Studies indicate that sanguinarine enhances the cytoplasmic superoxide dismutase immunoreactivity in broiler duodenal crypts. However, alkaloids, such as the aforementioned berberine, have low bioavailability in the body [150]. Consideration needs to be given to increasing its utilization through improved extraction processes, optimization of dosage forms, and selection of drug carriers.

## 7. Single and Compound Prescription of Herb with Therapeutic Properties for Oxidative Stress in the Intestinal Tract of Poultry

In recent years, significant progress has been made in the study of single and compound prescriptions of herbal medicines for combating intestinal OS. Table 3 condenses the latest research advances. However, due to the complexity of the components of single-flavored and compound herbal medicines, most studies have been confined to simple phenotypic observations and have not penetrated into the molecular mechanisms. Therefore, more in-depth studies should assess the efficacy of compound herbal medicines against intestinal oxidative stress.

## 8. Conclusions and Perspectives

In modern livestock production, intestinal health is one of the critical factors in ensuring animal growth and improving performance. OS is recognized as an essential cause of animal health, with excess free radicals leading to intestinal barrier damage, inflammatory responses, and impaired nutrient absorption, thereby affecting growth efficiency and overall health. Therefore, seeking effective interventions to alleviate intestinal oxidative stress is particularly important. Herbal medicines and their active ingredients are gradually becoming a potential option to address this issue due to their favorable antioxidant properties and safety. Although considerable progress has been made in research on natural drugs with antioxidant potential, these drugs should be studied in greater detail and more adequately before they are applied in poultry production to treat poultry intestinal OS.

The herbs and active ingredients discussed in this paper have only undergone preliminary pharmacological studies. The specific molecular mechanisms by which these herbs alleviate intestinal oxidative stress warrant further investigation in future research. Plant extracts are generally considered safer than certain chemical drugs, and consumers may be more confident in products that use plant-derived ingredients. Plant-derived drugs are often considered to be more environmentally friendly, which aligns with current consumer concerns about sustainability and eco-friendliness. However, from a consumer perspective, the decision to use plants or their extracts as medications to mitigate intestinal oxidative stress in poultry needs to weigh the advantages of natural ingredients against the uncertainty of effectiveness and cost. Providing transparent information and reliable evidence of efficacy will help increase consumer trust and acceptance. There is a notable lack of clinical data concerning the pharmacokinetics of the natural medicines identified and reported. Additionally, limited studies address the potential toxicity and side effects associated with prolonged or high doses of these herbal active ingredients in vivo. For instance, while *Galla Chinensis* has demonstrated positive effects on poultry gut development, it has also been regarded as an anti-nutritional factor in certain animals due to its capacity to precipitate essential nutrients [165]. This dual nature underscores the need for more comprehensive studies to assess these candidate herbs’ toxicity and side effects. Moreover, it is imperative to encourage further research that evaluates the practical effectiveness of these herbal candidates in production settings. Many herbal medicines exhibit low bioavailability when administered orally; thus, developing appropriate formulation types is essential. Establishing standardized production processes and quality control systems for herbal medicines is crucial to ensure their safety and efficacy. A thorough understanding of the potential applications of herbal medicine can be achieved through multidimensional, interdisciplinary, and integrated research approaches encompassing fields such as nutrition, toxicology, and epidemiology. By pursuing these avenues, we can enhance the scientific foundation supporting the use of herbal medicines in various therapeutic contexts.

Collectively, the present paper not only provides an overview of the mechanisms, factors, and negative impacts on intestinal health associated with the occurrence of intestinal OS in poultry but also summarizes the natural antioxidants, such as herbs and their active ingredients, that have been shown in recent years to have potential for preventing and treating intestinal OS. We hope that this review will guide the development of natural herbs and their extracts for treating oxidative stress in the poultry gut.

## Figures and Tables

**Table 1:** Effects of different types of feed toxins on the intestinal tract of poultry.

Types of Feed Toxins	Research Object	Dosage	Effects	Bibliography
Aflatoxin	Broiler	0.25 mg/kg in basal diet	Disrupted ileum architecture, diminished nutrient digestibility.	Alqhtani et al. [73]
	Broiler chicks	0.5 and 2 ppm	Increases in health enumerations of *Escherichia coli*, *Salmonella*, *Klebsiella*, and total negative bacteria, crypt depth, goblet cell counts, and lymphoid follicular diameter.	Jahanian et al. [74]
Deoxynivalenol	Broiler	10 mg/kg	Impaired the Na(+)-d-glucose cotransport in the jejunum.	Awad et al. [75]
	Broiler chickens	7.540 ± 2.20 mg/kg	Higher concentrations of mycotoxins in the distal portion of the small intestine, leading to impairment of the intestinal barrier.	Osselaere et al. [76]
Zearalenone	Broilers	1.59 mg/kg	Reduced numbers of duodenal CD4+ cells.	Revajová et al. [77]
T-2 toxin	Bobwhite quail	15 and 18 mg/kg BW	Marked lymphocyte necrosis and depletion throughout gut-associated lymphoid tissue in the small intestine were observed.	Grizzle et al. [78]
	Broiler chick	15.0 mg/kg diet	Decrease in propionic acid concentration and an increase in susceptibility to Salmonella colonization.	Kubena et al. [79]
Ochratoxin-A	Broiler chickens	50, 20 and 1 μg/kg/BW	Decreased body weight gain, poorer feed conversion ratio, lower leukocyte and lymphocyte count, and altered intestinal mucosa architecture.	Solcan et al. [80]
Fumonisin	Broiler	25.4 mg FB1 + FB2/kg feed	The intestinal mucus layer is altered.	Antonissen et al. [81]
Lead	Broiler chickens	200 mg lead acetate/kg of diet	The expression of all sugar, peptide, and amino acid transporters was significantly downregulated.	Ebrahimi et al. [82]
Cd	Broiler chickens	50 or 100 ppm	Reduction in leaf-like villi and increase in convoluted villi.	Teshfam et al. [83]

**Table 2:** In vitro experimental validation of plant extracts with therapeutic potential for oxidative stress.

Active Ingredients	Source	Dosage	Research Object	Oxidative Stressors	Effects	Signaling Pathway	Bibliography
Polysaccharide	*Astragalus membranaceus* extract	5–100 μg/mL	IEC-6 cells	LPS; IFN	Significantly reduced inflammatory response and pro-oxidant state	NF-κB, Nrf2	Adesso et al. [92]
	Fucoidan	0, 0.1, 1.0 or 2.5 mg/mL	Caco-2 cell	H_2_O_2_	Enhanced intestinal epithelial barrier function by upregulating the expression of claudin-1	N/A	Iraha et al. [93]
	Chinese yam *(Dioscoreae Rhizoma)* polysaccharide	200, 400 and 800 μg/mL	IEC-6 Cells	H_2_O_2_	Improved cell viability, SOD activity, and reduce intracellular ROS production and MDA content	MAPK	Li et al. [94]
	Longan (*Dimocarpus longan Lour.*) polysaccharides	2.5–40 μg/mL	IEC-6 cells	Fumonisin B1	Alleviates cellular oxidative stress by maintaining cellular barrier integrity	MAPK and mitochondrial apoptosis	Yu et al. [95]
	*Nelumbo nucifera* leaf polysaccharide	N/A	IPEC-J2 cells	H_2_O_2_	Increased the total cellular antioxidant capacity, antioxidant enzyme gene expression and reduced the accumulation of ROS	Nrf2	Huang et al. [96]
	Polysaccharides from red alga *Gelidium spinosum*	N/A	IEC-6 cells	H_2_O_2_	Obstructed oxidative stress in intestinal epithelial cells and hinders apoptosis	N/A	Ajala et al. [97]
Polyphenol	Water-soluble form of chestnut tannin	0.025, 0.05, 0.1and 0.2%	Chicken small intestinal epithelial cell	N/A	WST promotes enterocyte proliferation and maintains the antioxidant potential of the cells	N/A	Brus et al. [98]
	Wild blueberry (*V. angustifolium*)	1 and 5 mg mL^−1^	Caco-2 cell	TNF-α (10 ng mL^−1^)	Maintenance of cell barrier function by increasing claudin-1 expression and decreasing 8-OHdG	N/A	Marino et al. [99]
	Bioactive polyphenols from pomegranate (*Punica granatum* L.) Juice	10–1.25 μg/mL	IEC-6 cells	5-fluorouracil	Inhibited both inflammatory and oxidative stress parameters and apoptosis	N/A	Pepe et al. [100]
	Ginger essential oil, tea tree oil, grape seed extract, green tea extract, olive extract, pomegranate extract, chestnut extract, thyme essential oil, and *Capsicum* oleoresin	1, 10 and 100 ppm	Human Caco-2 cell	N/A	Control of intestinal barrier function and reduction of OS in intestinal epithelial cells	N/A	Toschi et al. [101]
	(Poly)phenol-rich extract derived from sweet cherry culls	50 μg·mL^−1^	Caco-2 cell	H_2_O_2_	Inhibit the formation or directly scavenge ROS-induced radicals	Nrf2	Matias et al. [102]
	Red raspberry (*Rubus idaeus*)	1–5 mg mL^−1^	Caco2 cell	IFN-γ and TNF-α	Attenuation of 8-OHdG levels and enhancement of claudin-1 expression	N/A	Marino et al. [103]
	Proanthocyanidin-rich grape seed extract	12.5 μg/mL	Caco-2 colon cells	LPS	The ROS-scavenging ability protects the intestinal lining from LPS-induced inflammation and restores the epithelial barrier integrity	N/A	Nallathambi et al. [104]
Saponin	Astragaloside IV	10 and 25 nM	Calf small intestine epithelial cells	H_2_O_2_	Protected calf small intestine epithelial cells, increasing cell survival, decreasing ROS generation and apoptosis	NFE2L2-antioxidant response element	Wang et al. [105]
	Ginsenoside Rf	N/A	Iintestinal epithelial cells (HT-29)	TNF-α/LPS	Rreduced the production of IL-1β, IL-6, TNF-α, NO and ROS	MAPKs/NF-κB	Ahn et al. [106]
	Compound K, a ginseng saponin metabolite	0–30 μM	ARPE-19 human retinal pigment epithelial cells	H_2_O_2_	Cytotoxicity, oxidative stress, DNA damage, mitochondrial impairment, and apoptosis were significantly attenuated	Nrf2	Park et al. [107]
	Astragaloside II	0.1 μM	Caco-2 cells	N/A	Promoted wound closure and increased cell proliferation, L-arginine uptake, CAT1 and CAT2 protein levels, total protein synthesis	mTOR	Lee et al. [108]
Flavonoids	Luteolin and chrysin	8.7 µM Luteolin, 1 µM Chrysin	IPEC-J2 cell line	LPS	Exerted beneficial effects on ROS levels and on cytokine secretion	N/A	Wohlert wt al. [109]
	Quercetin and its methylated derivatives 3-o-methylquercetin and rhamnazin	25 and 50 µM μg/mL	IPEC-J2 cell line	LPS	Decreased intracellular ROS production and prevented the disruption of confluent intestinal epithelial monolayer	N/A	Karancsi et al. [110]
	Anthocyanin-rich bilberry extract and resveratrol	1.25–20 μM	Caco-2 cells	Fatty acids and bile acids	Protected cells from cytotoxicity, intracellular ROS generation, and mitochondrial superoxide production	N/A	Ershad et al. [111]
	Flavonoid-rich concentrate recovered from *Opuntia ficus-indica* juice	40 and 160 μg/mL	Caco-2 cells	H_2_O_2_	Reduces NO and TNF-α expression and modulates apparent permeability	N/A	Matias et al. [112]
	Kaempferol	0, 5, 10 μmol L^−1^	IPEC-1 cells	Niquat	Maintains the intestinal barrier	Nrf2	Jin et al. [113]
	Total flavonoids of *Engelhardia roxburghiana* Wall. leaves	20, 40 and 60 μg/mL	HIEC-6 cells	Radiation	Reduced radiation-induced apoptosis and DNA damage and exerted radioprotective effects	Nrf2	Wu et al. [114]
Others	Organic acids from citric and sorbic acid	0.2 or 1 g/L	Caco-2 cells	N/A	Trans epithelial resistance was increased	N/A	Grilli et al. [115]
	Cinnamaldehyde	100 µM	IEC-6	*Porphyromonas gingivalis* derived lipopolysaccharide	Reducing ROS, MDA, and NO upregulated the expression of tight junction proteins	PI3K/Akt-Mediated NO/Nrf2	Sampath et al. [116]

Note: N/A means not involved.

**Table 3:** Antioxidant capacity of single and compound prescriptions of herbal medicines.

Herb	Collection Site	Form	Dosage	Animal	Oxidative Stressors	Effects	Signaling Pathway/Critical Factors	Bibliography
*Taraxacum mongolicum*	The dried whole herb *Taraxacum mongolicum* Hand.	Aqueous extract	500, 1000 and 2000 mg/kg	Broiler	N/A	Improvement of intestinal morphology, antioxidant status, immunity, and increase of dominant intestinal flora	SOD, GSH-Px, MDA, T-AOC, LZM, sIgA, TRF, ZO-1, and DEF β1	Dong et al. [151]
*Allium sativum* L.		Powder	1000 mg/kg	Broiler	LPS	Attenuated LPS-induced weight loss, OS and inflammation	TLR4/NF-κB	Zhang et al. [152]
*Origanum vulgare* L.		Essential oil	200 mg/kg	Broiler	N/A	Improved broiler growth performance, antioxidant activity and intestinal health, increased beneficial bacteria and digestive enzyme activity, and reduced harmful bacteria	SOD, GSH-Px, MDA, T-AOC	Zhang et al. [153]
*Astragalus membranaceus*	Root	Powder	5 g/kg	Broiler	N/A	Improvement of antioxidant capacity	SOD, GSH-Px, MDA	Zhang et al. [154]
*Lasia spinosa* Thw.	Root	Powder	1%, 2%, 4%	Guangxi partridge broilers	N/A	Improve growth performance, gut morphology, antioxidant capacity, calcium, magnesium, iron and phosphorus, and lowers blood lipids	CAT, GSH-Px, SOD	Zhang et al. [155]
*Phyllanthus emblica* L.	Leaves and branches mixture	Powder	1%, 2%	Broiler chickens	N/A	Reduction of harmful bacteria in the ileum and cecum, improved growth performance and further antioxidant effects	Nrf2-ARE	Lee et al. [156]
*Ginkgo biloba*	Leaves	Powder	1.5, 2.5, 3.5, 4.5 and 5.5 g/kg	Broilers	N/A	Enhancement of nutritional utilization and intestinal development in broilers by enhancing the activity of antioxidant enzymes, digestive enzymes and the expression of antioxidant enzyme genes	MDA, CAT, GSH-Px, T-AOC	Niu et al. [157]
Licorice and rutin	N/A	Powder	300 + 100 mg mixture/kg, diet; 300 + 200 mg mixture/kg diet, 600 + 100 mg mixture/kg, 600 + 200 mg mixture/kg diet	Quails	N/A	Improvement of production performance, egg quality and antioxidant capacity, alteration of microbial composition	SOD, MDA	Li et al. [158]
A mixture of olive, laurel, and rosemary leaf powders	Leaves	Powder	6 g/kg	Laying hens	N/A	Elevated oxidative status, biochemical, immunological, intestinal morphophysiological parameters and egg quality	SCFAs, TOS, ROMs, IL-6, IL-1β, and TNF-α	D’Alessandro et al. [159]
*Astragalus membranaceus* and *Codonopsis pilosula*	N/A	N/A	500 mg/kg	Broiler	N/A	Changed the composition of gut microbiota, improved feed utilization	Claudin-1, IL-6, IFN-γ, IFN-β, TNF-α, IL-10,	Liu et al. [160]
Rougan decoction (*Hypericum japonicum, Alisma orientalis, Paeonia lactiflora,* Poria, Glycyrrhizae Radix*, Schisandra chinensis* fruit*, and Magnolia* of*ficinalis*)	N/A	The water extraction and alcohol precipitation method	N/A	Chicken	LPS	Reduced oxidative damage in chicken hepatocytes	Nrf2/ARE	Wang et al. [161]
*Portolaca oleracea* L., Sophorae Flavescentis Radix, *Thalictrum glandulosissimum, Terra flava usta,* and *Pogostemon cablin*	N/A	Powder	1000 mg/kg	Yellow-feathered broilers	N/A	Improved yellow-feathered broilers’ growth performance and immune status	CLDN-1, OCLN, ZO-1, SOD, GSH-Px, T-AOC, CAT	Liu et al. [162]
*Astragalus membranaceus*, tangerine peel, dandelion and thyme	N/A	Powder	0.25%, 0.5%, 1%, 2%	Brolier	N/A	Promoted the growth of small intestinal villi, increase the VH/CD ratio in all intestinal segments, and enhanced the antioxidant property of the body	T-AOC, CAT, SOD, occludin, claudin-1	Fu et al. [163]
Licorice, *Codonopsis pilosula, Astragalus membranaceus, Atractylodes macrocephala* Koidz., *Angelica sinensis*, *C*imicifugae Rhizoma, Bupleuri Radix*,* dried tangerine peel, ginger, and jujube	Root	Powder	0.2, 0.4, 0.6, 0.8%	Ningdu yellow chickens	N/A	Enhancement of immune and antioxidant functions, improvement of jejunal morphology and positive effects on cecum microbiota	SOD, GSH-Px, T-AOC, CAT	Song et al. [164]

Note: N/A means not involved.

## Data Availability

All data are presented in this article.
